# Seasonal shifts in accumulation of glycerol biosynthetic gene transcripts in mountain pine beetle, *Dendroctonus ponderosae* Hopkins (Coleoptera: Curculionidae), larvae

**DOI:** 10.7717/peerj.3284

**Published:** 2017-06-13

**Authors:** Jordie D. Fraser, Tiffany R. Bonnett, Christopher I. Keeling, Dezene P.W. Huber

**Affiliations:** 1 Ecosystem Science and Management Program, University of Northern British Columbia, Prince George, British Columbia, Canada; 2 Department of Biological Sciences, Simon Fraser University, Burnaby, British Columbia, Canada; 3 Michael Smith Laboratories, University of British Columbia, Vancouver, British Columbia, Canada

**Keywords:** Bark beetle, Mountain pine beetle, Overwintering, Glycerol, Seasonal, Cyroprotectant, Freeze intolerant, *Dendroctonus ponderosae*, Coleoptera, Curculionidae

## Abstract

Winter mortality is a major factor regulating population size of the mountain pine beetle, *Dendroctonus ponderosae* Hopkins (Coleoptera: Curculionidae). Glycerol is the major cryoprotectant in this freeze intolerant insect. We report findings from a gene expression study on an overwintering mountain pine beetle population over the course of 35 weeks. mRNA transcript levels suggest glycerol production in the mountain pine beetle occurs through glycogenolytic, gluconeogenic and potentially glyceroneogenic pathways, but not from metabolism of lipids. A two-week lag period between fall glycogen phosphorylase transcript and phosphoenolpyruvate carboxykinase transcript up-regulation suggests that gluconeogenesis serves as a secondary glycerol-production process, subsequent to exhaustion of the primary glycogenolytic source. These results provide a first look at the details of seasonal gene expression related to the production of glycerol in the mountain pine beetle.

## Introduction

While native to British Columbian forests, the mountain pine beetle, *Dendroctonus ponderosae* Hopkins (Coleoptera: Curculionidae), population sizes have reached epidemic levels, resulting in the largest infestation on record (Westfall, 2007), causing significant economic and social impact in forestry dependent communities. Winter cold temperatures are often cited as the largest single source of mortality in *D. ponderosae* (Safranyik, 1978; Cole, 1981; Safranyik & Carroll, 2006; Stahl, Moore & McKendry, 2006; Aukema et al., 2008). While fall and winter temperatures regularly reach far below the equilibrium freezing point of mountain pine beetle bodily fluids, larvae are able to avoid the damaging effects of ice formation.  The phenomenon of quiescence (Powell & Logan, 2005) grants overwintering mountain pine beetle larvae the ability to reallocate limited energy reserves from developmental and basal metabolism toward biosynthesis of antifreeze compounds (Li, Ding & Goto, 2002; Joanisse & Storey, 1994b).

**Figure 1:**
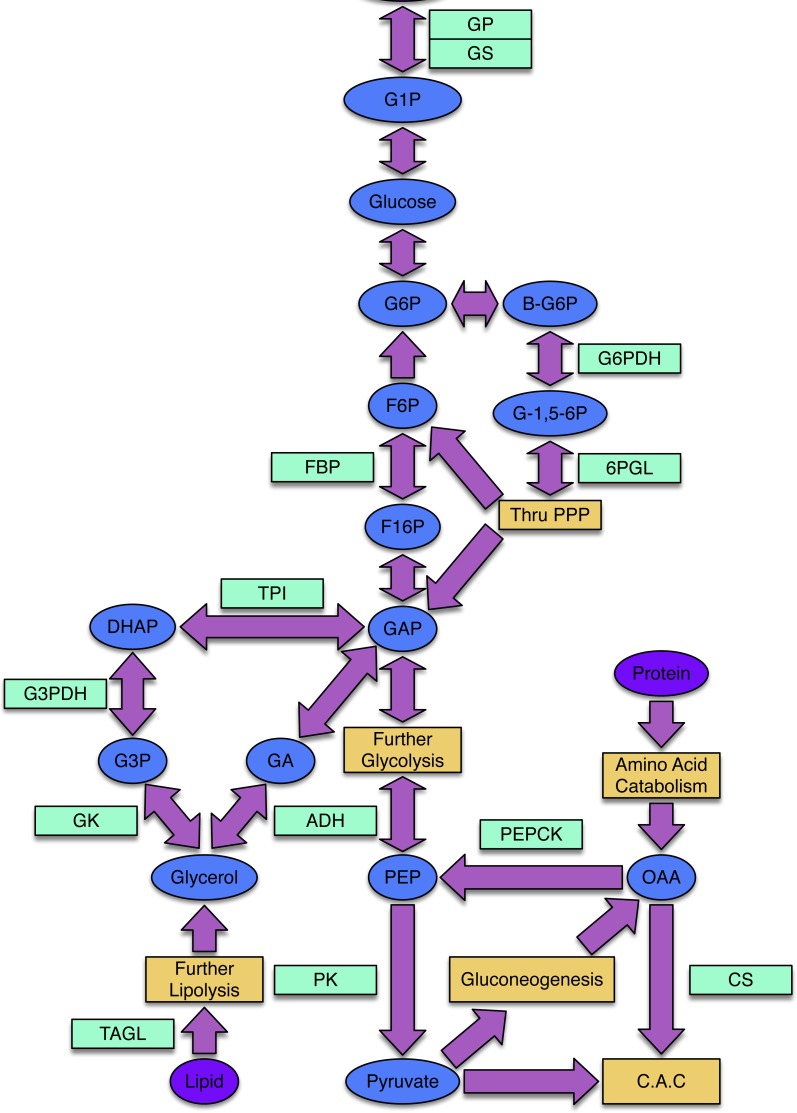
The processes of glycogenesis and glycogenolysis involve the following enzymes: glycogen phosphorylase (GP); triosephosphate isomerase (TPI); glycerol-3-phosphate dehydrogenase (G3PDH); alcohol dehydrogenase (ADH); glycogen synthase (GS). The metabolism of lipids, specifically triglycerides, produces glycerol and occurs via triacylglycerol lipase (TAGL). Reducing equivalents required in the production of glycerol can be produced via the pentose phosphate pathway (PPP), involving the enzymes 6-phosphoglucolactonase (6-PGL) and glucose-6-phosphate dehydrogenase (G6PDH). The enzyme citrate synthase (CS) is involved in the citric acid cycle (CAC) and gluconeogenic enzymes include phosphoenolpyruvate carboxykinase (PEPCK) and fructose-1,6-bisphosphatase (FBP). Pyruvate kinase (PK) is an enzyme present within the lower-half of glycolysis. The following substrates are involved in the above processes: glucose-1-phosphate (G1P), glucose-6-phosphate (G6P), β-glucose-6-phosphate (β-G6P), glucono-1,5-lactone 6-phosphate (G-1,5-6P), fructose-6-phosphate (F6P), fructose-1,6-bisphosphtate (F16P), dihydroxyacetone phosphate (DHAP), glycerol-3-phosphate (G3P), glyceraldehyde-3-phosphate (GAP), glyceraldehyde (GA), phosphoenolpyruvate (PEP) and oxaloacetate (OAA).

Freeze avoidant insects, like the mountain pine beetle, evade cold mortality by producing cryoprotectants, often polyols, that alter the freezing properties of their bodily fluids (Bale, 2002; Baust, 1983). Glycerol is the most common cryoprotectant used by insects to achieve states of freeze avoidance (Storey & Storey, 2004). While little work has been done to illuminate mechanisms by which the mountain pine beetle achieves a state of cold tolerance, Bentz & Mullins (1999) assessed the composition and seasonal quantity of polyols in mountain pine beetle hemolymph and showed that glycerol is the most abundant cryoprotectant accumulated by overwintering larvae. The metabolic pathways that lead to glycerol production in insects and other animals are well understood (Fig. 1).

The precise mechanisms by which mountain pine beetle larvae produce glycerol and the timing of production are unknown. Because glycerol is important in cold tolerance physiology in the mountain pine beetle, a better understanding of the dynamics of gene expression related to glycerol biosynthesis is of significant ecological importance, particularly as the insect moves into a new, and colder, habitat (Cullingham et al., 2011; Janes et al., 2014).

The objective of this study was to document seasonal gene expression differences in the mountain pine beetle for genes associated with glycerol production by assessing changes in transcript levels for genes in that biosynthetic pathway.

## Materials and Methods

### Sample and temperature collection

Eleven lodgepole pine (*Pinus contorta*) trees that had been attacked by mountain pine beetles in the previous summer —located west of Tête Jaune Cache, British Columbia, Canada (N53°3′35.28″*W*119°36′52.74″) —were sampled in 2008 and 2009. Three temperature data loggers (iButton^®^ data loggers; Maxim, Sunnyvale, CA, US) were affixed to each tree—at the base of the tree, at breast height on the north side of the tree, and at breast height on the south side of the tree. Temperature data were recorded every 30 min throughout the study period: September 19, 2008 through May 13, 2009. Due to technical difficulties, seventeen hours of temperature data were lost between 9:36 a.m., 1 April, 2009 and 2:36 a.m., 2 April, 2009. Daily minimum, mean and maximum temperatures for 1 April, 2009 were estimated by averaging daily minimum, mean and maximum temperatures for 31 March, 2009 and 2 April, 2009.

Multiple live mountain pine beetle larvae of mixed instar and undetermined sex were manually removed from trees on the following dates: 19 September, 2008, 3 October, 2008, 17 October, 2008, 31 October, 2008, 14 November, 2008, 18 March, 2009, 1 April, 2009, 14 April, 2009, 29 April, 2009 and 13 May, 2009. Larvae were individually deposited in 1.5mL microcentrifuge tubes, immediately flash frozen in liquid nitrogen, and were transported back to UNBC laboratory facilities on dry ice where they were immediately stored at −80 °C until RNA extractions were conducted.

### RNA isolation and cDNA synthesis

Larval samples were incubated for 18 h in RNAlater^®^-ICE (Ambion, Austin, TX, USA) at −20 °C and completely homogenized by use of a GeneoGrinder 2000 (SpexCertiprep, Metuchen, NJ, USA). RNA extractions were conducted with MagMax™-96 Kits (Ambion, USA) containing a DNase digestion step. RNA concentration was acquired by use of a Qubit Quantification System (Invitrogen, Carlsbad, CA, USA). Estimates of sample purity were obtained by use of a Nanodrop ND-1000 (NanoDrop Technologies, Inc., Wilmington, DE, USA), with sample 260/280 ratios ranging from 1.8–2.2. Sample integrity was assessed via Experion StdSens (BioRad, USA) microfluidics chips. RNA aliquots were assessed for genomic DNA contamination via RT-qPCR, ensuring a minimum difference of 5 *C*_q_ between RNA and cDNA runs for the same biological sample (Nolan, Hands & Bustin, 2006). RNA samples were stored at −80 °C until reverse transcription reactions were conducted. Sample cDNA was produced from 800 ng of total RNA using random decamers and the High Capacity cDNA Reverse Transciption Kit (Invitrogen, Carlsbad, CA, USA) in a total reaction volume of 40 µL following the manufacturer’s protocol.

### RT-qPCR target oligonucleotide information and protocol

Candidate gene sequences used for the identification of transcripts and proteins of glycerol biosynthesis were identified from previously developed mountain pine beetle EST and full-length cDNA databases (Keeling et al., 2012). We investigated differential transcript accumulation for the following mountain pine beetle gene sequences: pyruvate kinase (*PK*, BT127907), glycogen phosphorylase (*GP*, APGK01006417), citrate synthase (*CS*, APGK01047658), 6-phosphoglucolactonase (*6-PGL*, BT128455), glucose-6-phosphate dehydrogenase (*G6PDH*, APGK01024792), glycerol-3-phosphate dehydrogenase (*G3PDH*, BT128609), fructose-1,6-bisphosphatase (*FBP*, BT128229), alcohol dehydrogenase (*ADH*, BT127435), triosephosphate isomerase (*TPI*, BT127767), phosphoenolpyruvate carboxykinase (*PEPCK*, BT127980), glycogen synthase (*GS*, APGK01035513) and triacylglycerol lipase (*TAGL*, BT127387). Primer sequences and gene-specific properties are shown in Table 1. Hydrolosis probes were designed for each of the genes of interest and used TAMARA, ROX or FAM fluorophores (Table 2).

**Table 1:** Sequences and properties of primers used in evaluation of twelve genes of interest employed to investigate seasonal cold tolerance in *D. ponderosae*.

Reference Gene		Sequence (5′–3′)	(Primer) nM	% GC	T_*A*_	Amplicon size (bp)	DLR	*E*	*R*^2^
*PK*	Pyruvate kinase	CTTATCCTTTGGCTATTGCTTTGG	600	41.6	62.5	123	24 pg –75 ng	91.5	0.996
		ATCTGTGGTCAGCTTAATAGTATCG	300	40	62.5				
*GP*	Glycogen phosphorylase	TGGATCAAATGCAGAACGGATTC	600	43.4	63.6	107	5 pg –75 ng	94.9	0.999
		GTAATCGGCCAGCAAGAAGAAC	600	50.0	63.6				
*CS*	Citrate Synthase	GACTTCGATTTGTGACGAGAGAG	600	47.8	63.0	142	12 pg –75 ng	93.9	0.999
		CAGACGTATGGAGGCAAACATC	300	50	63.0				
*6-PGL*	6-phosphoglucolactonase	CCGATTTGATCTACTGCTGCTG	600	50	53.8	108	12 pg –75 ng	98.9	0.997
		GTGATTGGAGCCACCCATTTG	900	52.3	53.8				
*G6PDH*	Glucose-6-phosphate dehydrogenase	GCAGAAGTAAGAATTCAGTTTGAGG	900	40	62.5	137	5 pg –75 ng	100.1	0.995
		GCCATACCAGGAGTTTTCACC	900	52.3	62.5				
*G3PDH*	Glycerol-3-phosphate dehydrogenase	TGTTCTGCGAAACCACCATTG	900	47.6	53	126	24 pg –75 ng	95.5	0.999
		CGCCGCAAACTTCCACAG	300	61.1	53				
*FBP*	Fructose-1,6-bisphosphatase	CACAGCTACCGGAGAACTCAC	300	57.1	63.3	139	24 pg –75 ng	96.9	0.999
		CACTTCTTCGCCCTGTACATTTG	900	47.8	63.3				
*ADH*	Alcohol dehydrogenase	ATCCTCTACACGGCGGTTTG	900	55	63.3	147	12 pg –75 ng	90	0.996
		ATCACCTGGCTTCACACTGG	300	55	63.3				
*TPI*	Triosephosphate isomerase	ACGCCCCAGCAAGCTCAG	900	66.6	63.3	106	24 pg –75 ng	96.3	0.998
		CCGAACCGCCGTATTGGATTC	900	57.1	63.3				
*PEPCK*	Phosphoenolpyruvate carboxykinase	GGCATCGAACTCACTGACTCC	600	57.1	63.0	129	5 pg –75 ng	100	0.995
		GGTGCCGACCGAGTGGAG	300	72.2	63.0				
*GS*	Glycogen synthase	GAACGACCCGGTGCTCAG	300	66.6	57.8	125	24 pg –75 ng	92.9	0.996
		CGTAGTCCAGCCCGAAGAG	600	63.1	57.8				
*TAGL*	Triacylglycerol lipase	TCTACGTGTATACCTCTCAGAATCG	300	44	63.0	100	24 pg –75 ng	92.8	0.999
		GTGTCTCTGTTAGCAGCGAATC	600	50	63.0				

**Table 2:** Sequences of hydrolysis probes used in evaluation of twelve genes of interest employed to investigate seasonal cold tolerance in *D. ponderosae*.

Gene		Probe sequence (5′–3′)
*PK*	Pyruvate kinase	56-TAMN-CAGCAGATCCTCCGCCTTCCAACAA-3BHQ_2
*GP*	Glycogen phosphorylase	56-ROXN-CAGCCCAAGCAATCCAGACGAGTTC-3BHQ_2
*CS*	Citrate synthase	56-FAM-CCACAGCAACGAAATAACACCACCA-3BHQ_1
*6-PGL*	6-phosphoglucolactonase	56-TAMN-ACACCTGCTCTCTGTTTCCTGGACA-3BHQ_2
*G6PDH*	Glucose-6-phosphate dehydrogenase	56-ROXN-AGCCTCGCCTGGTTGAACTCTAATC-3BHQ_2
*G3PDH*	Glycerol-3-phosphate dehydrogenase	56-TAMN-TCATCGTCCACCACCACCACTCG-3BHQ_2
*FBP*	Fructose-1,6-bisphosphatase	56-ROXN-AGCTACTCAATGCCATCCAGACTGC-3BHQ_2
*ADH*	Alcohol dehydrogenase	56-FAM-TCCAACACTCTCGACCACTCCAGC-3BHQ_1
*TPI*	Triosephosphate isomerase	56-TAMN-AAGTCCATCAGTCGCTACGCCAGTG-3BHQ_2
*PEPCK*	Phosphoenolpyruvate carboxykinase	56-ROXN-TTGACGAACTCCTCCGCCTCTTGC-3BHQ_2
*GS*	Glycogen synthase	56-TAMN-TCTTCAACACCGCCGAGGACCG-3BHQ_2
*TAGL*	Triacylglycerol lipase	56-ROXN-ACCCAAATAAGAGCCAGTGACGCCA-3BHQ_2

All reactions were conducted on an iQ5 (Bio-Rad, Hercules, CA, USA) real-time quantitative PCR machine and consisted of a denaturation step at 95 °C for 3 min followed by 40 cycles of 10 s at 95 °C and 30 s at each gene of interest’s specific annealing temperature (Table 1). cDNA for each sample was diluted 1:9 with nuclease-free water and was run in duplicate as a technical replicate. Reaction volumes consisting of 25 µL total volume consisting of the following component volumes were conducted: 2.5 µL forward primer (9 µM), 2.5 µL reverse primer (9 µM), 2.5 µL Probe (2.5 µM), 2.5 µL cDNA template, 2.5 µL nuclease-free water and 12.5 µL iQ Supermix (2X; Biorad, Hercules, CA, USA). No template controls were utilized for all reactions. Gene transcript accumulation values were obtained from 4-8 larval biological replicates, collected from eleven separate lodgepole pine trees.

**Figure 2:**
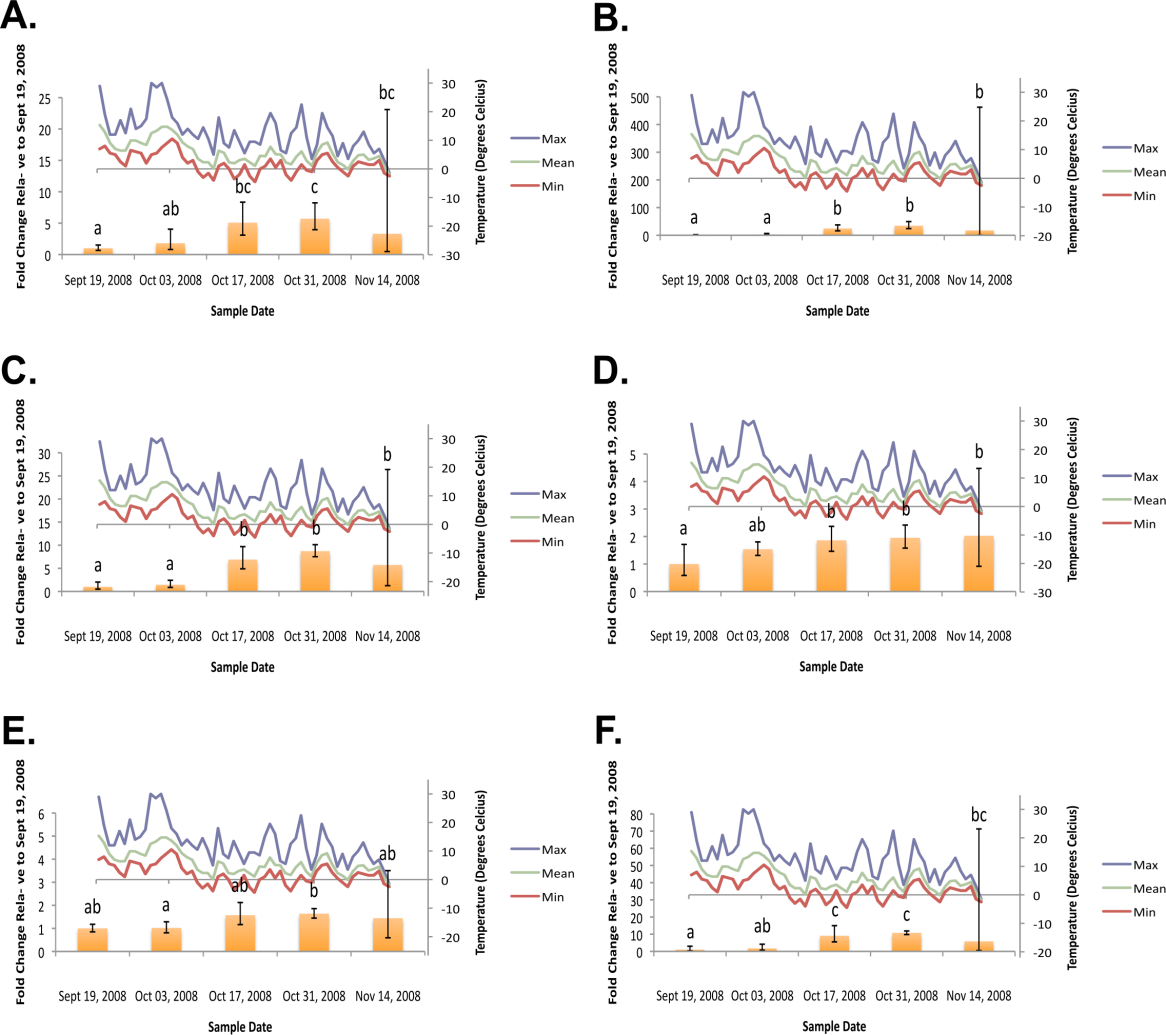
Geometric mean fold change in mountain pine beetle transcript accumulation relative to 19 September, 2008 and corresponding daily seasonal thermal data at the larval collection site for the fall 2008 study period. (A) Glycogen phosphorylase relative mRNA expression (Fall 2008); (B) Phosphoenolpyruvate carboxykinase relative mRNA expression (Fall 2008); (C) fructose-1,6,-bisphosphatase relative mRNA expression (Fall 2008); (D) Glycogen synthase relative mRNA expression (Fall 2008); (E) triose-phosphate isomerase relative mRNA expression (Fall 2008); (F) glyerol-3-phosphate dehydrogenase relative mRNA expression (Fall 2008). Gene transcript accumulation values were obtained from 4–8 larval biological replicates, collected from eleven separate lodgepole pine trees, with 95% confidence intervals being displayed. One-way ANOVA were conducted for transcript accumulation data followed by Tukey’s HSD post-hoc test for pair-wise multiple comparisons. Means found to be statistically different (*p* < 0.05) are denoted with different lowercase letters.

**Figure 3:**
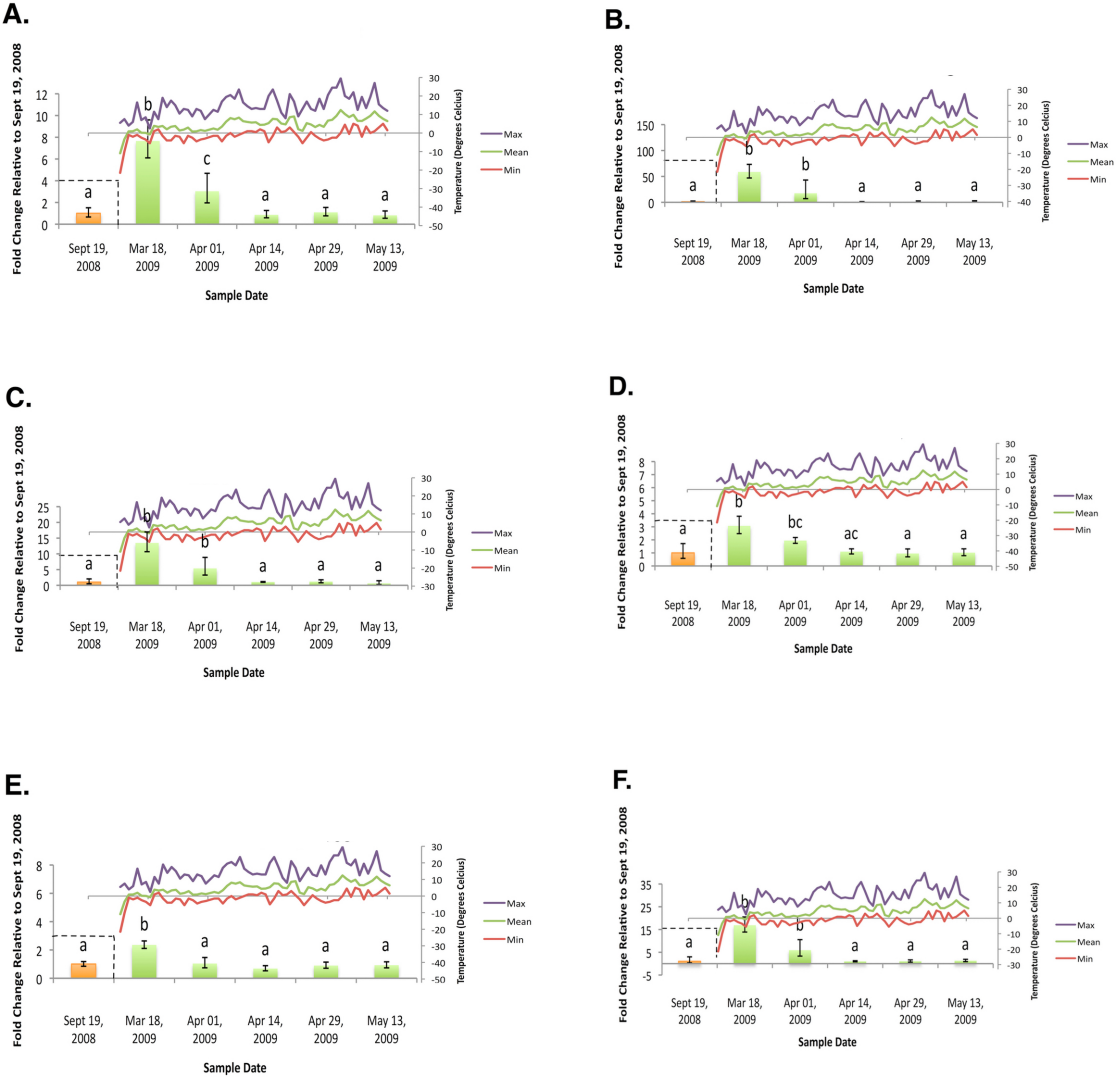
Geometric mean fold change in mountain pine beetle transcript accumulation relative to 19 September, 2008 and corresponding daily seasonal thermal data at the larval collection site for the spring 2009 study period. (A) Glycogen phosphorylase relative mRNA expression (Spring 2009); (B) Phosphoenolpyruvate carboxykinase relative mRNA expression (Spring 2009); (C) fructose-1,6,-bisphosphatase relative mRNA expression (Spring 2009); (D) Glycogen synthase relative mRNA expression (Spring 2009); (E) triose-phosphate isomerase relative mRNA expression (Spring 2009); (F) glyerol-3-phosphate dehydrogenase relative mRNA expression (Spring 2009). Gene transcript accumulation values were obtained from 4–8 larval biological replicates, collected from eleven separate lodgepole pine trees, with 95% confidence intervals being displayed. One-way ANOVA were conducted for transcript accumulation data followed by Tukey’s HSD post-hoc test for pair-wise multiple comparisons. Means found to be statistically different (*p* < 0.05) are denoted with different lowercase letters.

### Data analysis

Transcript accumulation normalization for each biological replicate was achieved from a normalization factor made up of: RNA polymerase II (*RPII*, BT126845), porphobilinogen deaminase (*PBD*, GAFW01009520), actin (*ACT*, BT126695), tyrosine 3-monooxygenase (*YWHAZ*, BT128603) reference gene transcript accumulation data. These four reference genes were determined by geNorm analysis (Vandesompele et al., 2002) to be most appropriate for transcript accumulation normalization consisting of biological replicates from all treatment groups (i.e., all ten data time points). For statistical analysis of each gene of interest, logarithmically transformed normalized mRNA expression data for biological replicates were analyzed in R (Version 2.9.2). Analysis of variance (ANOVA) assumptions of homoscedasticity (Levene’s test) and normality (histograms, quantile–quantile plots, the Sahpiro-Francia normality test) were assessed for mRNA expression data. One-way ANOVAs were conducted for mRNA expression data followed by Tukey’s HSD *post-hoc* test for pair-wise multiple comparisons. For graphical analyses, data normalization was performed as in Willems, Leyns & Vandesompele (2008). Geometric mean fold changes for each treatment were set relative to 19 September, 2008 mean mRNA expression level. Relative fold changes and their respective 95% confidence intervals were plotted in Microsoft Excel.

## Results and Discussion

### RT-qPCR target gene expression results

In the fall (collection dates between September and November), statistically significant increases in transcript levels were observed for GP, PEPCK, FBP, GS, TPI, and G3PDH (Fig. 2). In the spring (collection dates between March and May), statistically significant decreases in transcript levels were observed for GP, PEPCK, FBP, GS, TPI and G3PDH (Fig. 3). TAGL, ADH, 6-PGL, G6PDH, PK and CS transcript levels did not exhibit differential accumulation during either the fall or the spring study periods (not statistically significant, data not shown). When compared and contracted to proteomic data available for the same sample population, our results mirror quite closely (Bonnett et al., 2012).

### Transcript accumulation indicates that glycerol biosynthesis involves both glycogenolysis and gluconeogenesis

One of the predominant carbohydrate energy reserves used by overwintering insects is glycogen (Li, Ding & Goto, 2002; Han & Bauce, 1998; Klowden, 2002), whereas the most common lipid store comes in the form of triglycerides (Klowden, 2002). Because they do not feed during the winter (Régnière & Bentz, 2007; J Fraser, pers. obs., 2008), mountain pine beetle larvae are in a state of near-starvation and must efficiently allocate limited energetic stores between maintaining basal metabolic levels and producing cryoprotectants, mainly glycerol (Bentz & Mullins, 1999). Measures of how overwintering metabolic rates vary and which energetic substrates are consumed in the production of glycerol have yet to be obtained for the mountain pine beetle. As both glycogen and triglycerides have the potential to be converted into glycerol (Fig. 1), genes involved in both glycogenolysis (*GP*) and lipolysis (*TAGL*) were selected for investigation.

There was no significant differential transcript accumulation for *TAGL* in either fall or spring study periods. This result does not support the hypotheses that glycerol is produced from the metabolism of triglycerides, and that triglycerides are used as an energy source for the mountain pine beetle post-quiescence. This finding is contrary to that of a study of the closely related pine engraver *Ips pini* Say (Coleoptera: Curculionidae) (Lombardero et al., 2000) which indicated that lipids were the source of overwintering glycerol metabolism. Mixed evidence has been generated from similar metabolite assays conducted within the goldenrod gall fly (*Eurosta solidaginis*). Early observations found a concomitant decline in lipid content and an increase in glycerol accumulation (Morrissey & Baust, 1976), but subsequent studies observed stable overwintering lipid levels for this same insect species (Storey & Storey, 1986), which mirrors our findings for *TAGL* expression. Furthermore, seasonal proteomic data obtained from the same population of mountain pine beetles sampled for this study (Bonnett et al., 2012), specifically results for the LSD1 protein –responsible for activating triglyceride breakdown –added additional support our *TAGL* findings.

During the fall study period a statistically significant increase in *GP* transcript accumulation from 19 September to 17 October was observed and corresponded to a substantial decrease in temperature. Conversely, during the spring study period, a statistically significant decrease in *GP* transcript levels occurred from 18 March to 1 April and corresponded with a substantial increase in temperature. The opposing trends observed between *GP* transcript accumulation and temperature in both the fall (Fig. 2A) and the following spring (Fig. 3A) support the hypothesis that glycerol production is the result of the metabolism of glycogen in the mountain pine beetle. This conclusion is similar to the findings of numerous other studies of glycerol accumulation in overwintering insects, including: metabolite assays observing glycogen depletion (Storey, Baust & Storey, 1981a; Storey & Storey, 1986; Pullin & Bale, 1989; Churchill & Storey, 1989; Han & Bauce, 1998; Li, Ding & Goto, 2002); increased glycogenolytic enzyme activities (Storey & Storey, 1981b; Joanisse & Storey, 1994a; Joanisse & Storey, 1995; Clow, Ewart & Driedzic, 2008); and increased glycogenolytic gene transcript accumulation (Richards et al., 2010). Transcript accumulation data reported for *GP* herein was found to be consistent with the proteomic findings of Bonnett et al. (2012).

In the fall study period a statistically significant increase in *PEPCK* transcript accumulation from 3 October to 17 October was observed and corresponded with a substantial decrease in temperature. Conversely, in the spring study period a statistically significant decrease in *PEPCK* transcript accumulation occurred from 1 April to 14 April and corresponded with a substantial increase in temperature. The opposite trends observed between temperature and *PEPCK* transcript accumulation for both the fall (Fig. 2B), and spring (Fig. 3B) study periods supports the hypothesis that, in addition to glycogenolysis, gluconeogenesis contributes to the production of overwintering glycerol in mountain pine beetle larvae. This hypothesis is further supported by proteomic findings for overwintering mountain pine beetles from the same population (Bonnett et al., 2012).

A two-week lag period between fall *GP* and *PEPCK* transcript up-regulation suggests that gluconeogenesis could serve as a secondary source for glycerol production subsequent to the potential exhaustion of the primary glycogenolytic source; a successive “one-two” punch of glycerol production. Further pieces of supporting evidence include the comparative intensities at which *GP* mRNA and *PEPCK* mRNA up-regulation occur. Between 3 October and 17 October, *GP* relative fold-change values increased more than 282%. Over this same period of time, *PEPCK* relative fold-change values increased by 919%. Where *GP* mRNA up-regulation appears to be quite gradual over a four-week period, *PEPCK* mRNA up-regulation displays a much larger fold-change increase over a shorter (two week) period of time.

Increased PEPCK activity has been reported in cultured hepatocytes from fasting mammals (Azzout et al., 1986) and fasting mammalian liver tissues (Hagopian, Ramsey & Weindruch, 2008). Gluconeogenesis has been observed in other species of insects post-fasting (Zhou et al., 2004) and it is possible that increased *PEPCK* transcript accumulation in the mountain pine beetle larvae is induced by experiencing near-starvation during the winter in conjunction with, or even in spite of, declining fall temperatures.

Further supporting the hypothesis that gluconeogenesis is induced in overwintering mountain pine beetle larvae are the seasonal *FBP* transcript accumulation results (Fig. 2C and Fig. 3C). FBP catalyzes reactions downstream of a mechanism branch point that can route carbon produced from the catabolism of amino acids to glycerol production (Fig. 1). The *FBP* seasonal transcript accumulation profile produced for mountain pine beetle larvae suggests that, in addition to glycerol production, gluconeogenesis may produce additional glucose as well. In a similar way, in addition to producing glycerol, cold treatment of rainbow smelt hepatocypes also produced glucose (Clow, Ewart & Driedzic, 2008).

### Transcript accumulation dynamics indicate that glycerol is not converted to glycogen by glycogenesis

We observed the transcript levels of genes associated with glycerol consumption by mountain pine beetles after spring temperatures had increased and cryoprotection was no longer essential. Our results indicate that a negative relationship exists between spring glycogen synthase (*GS*) transcript accumulation and temperature, a finding similarly observed in proteomic data from the mountain pine beetle (Bonnett et al., 2012). These results fail to support the hypothesis that glycerol is converted to glycogen in the spring (Fig. 2D). We did not expect to observe similar *GS* transcript profile and *GP* transcript accumulation profiles, as these two enzymes catalyze competing glycogenolytic and glycogenic reactions (Fig. 3D).

### Transcript accumulation dynamics indicate that glycerol is metabolized from a DHAP intermediate and by glyceroneogenesis

Within arthropods there has been much debate about from which intermediary substrates glycerol is produced (Storey, 1997). Consensus has centered around two triose-phosphate substrates, both produced in the process of glycogenolysis: dihydroxyacetone phosphate (DHAP) and glyceraldehyde-3-phosphate (GAP) (Fig. 1).

Findings from *in vivo* studies comparing enzyme activity levels for G3PDH and G3Pase versus GAPase and ADH in the Asiatic rice borer (Li, Ding & Goto, 2002) and the goldenrod gall fly (Joanisse & Storey, 1994a) support the metabolism of GAP, versus DHAP, as a glycerol intermediate. Accumulation of G3P during the cessation of glycerol synthesis (Storey, Baust & Storey, 1981a) and increased transcript accumulation of *G3PDH* both prior and during glycerol production (Liebsher et al., 2006; Richards et al., 2010) present the metabolism of DHAP as plausible source of glycerol synthesis.

While no differential transcript accumulation was observed in the fall for *ADH* transcript (not statistically significant, data not shown), a seasonal decline in transcripts was detected for *TPI* (Fig. 2E) and *G3PDH* (Fig. 2F). These results support the hypothesis that glycerol is metabolized from a DHAP intermediate rather than from a GAP intermediate in the mountain pine beetle.

Glyceroneogenesis is an abbreviated form of gluconeogenesis that leads from the catabolism of amino acid precursors to produce G3P (Hanson & Reshef, 2003). Shown previously to be important in fat metabolism in other insects (Okamura et al., 2007), glyceroneogenesis, like gluconeogenesis, is highly regulated by the transcript accumulation of *PEPCK*. Fold-change increases for *PEPCK* mRNA in mountain pine beetle larvae during periods of cold exposure were far greater than any other gene investigated within this study, reaching a high of 58.64-fold 18 March. This increased *PEPCK* transcript accumulation is consistent with patterns expected during periods of cold-induced glyceroneogenesis.

As was indicated from the aforementioned *GS* transcript accumulation analysis (Fig. 3D), when spring temperatures increase and cryoprotectant reserves are no longer essential to maintain, it is likely that the larvae do not reconvert glycerol into glycogen. Others have hypothesized that glycerol is metabolized through the citric acid cycle (Storey & Storey, 1986; Joanisse & Storey, 1994a). We did not observe mRNA up-regulation during the spring study period for *CS* (an enzyme involved in the citric acid cycle); *PK* (a required glycolytic enzyme); or enzymes from either of the two possible glycerol producing pathways (*ADH*, *G3PDH*, and *TPI*) which catalyze reversible glycerol metabolizing reactions. Failure to observe mRNA transcript accumulation for these genes supports the hypothesis that glycerol is metabolized by means other than via citric acid cycle.

### Transcript accumulation dynamics indicate that glycerol production does not involve the pentose phosphate pathway

Insect glycerol production involves a flux between oxidizing (i.e., NAD^+^ or NADP^+^) and reducing (i.e., NADH or NADPH) equivalents (Meyer, 1978; Wood & Nordin, 1980; Tsumuki et al., 1987; Storey & Storey, 1990). However differential transcript accumulation for two key PPP enzymes—*6PGL* and *G6PDH*—was not observed in mountain pine beetle larvae during the time course of our study. While our results do not support the hypothesis that the PPP is involved in the production of glycerol in the mountain pine beetle, regulation of enzyme function could instead be accomplished at the protein activity control level as observed in other insect species (Joanisse & Storey, 1995; Kostal et al., 2004) or through other means, such as developmental state, diapause transition, or phosphorylation state of enzymes (e.g., Li, Ding & Goto, 2002; Joanisse & Storey, 1994a; Joanisse & Storey, 1995).

## Conclusion

Differential transcript accumulation of important glycerol biosynthetic pathway genes in overwintering mountain pine beetle larvae support the hypothesis of glycerol production through glycogenolytic, gluconeogenic, and potentially glyceroneogenic pathways, but not through lipolytic means. Aerobic metabolism, as indicated by activity within the citric acid cycle, seems to remain constant during periods of increased glycerol production. The PPP appears to be potentially uninvolved with glycerol production, and an alternative source for reducing equivalents may exist. Transcript accumulation results for *TPI* and *G3PDH* along constant expression results for *ADH* support the hypothesis that glycerol is produced from a DHAP, versus a GAP, intermediate. Glycogenesis does not appear to occur in the spring when glycerol is no longer needed as a cryoprotectant for larvae. Our gene transcript results closely mirror proteomic data produced from the same sample population (Bonnett et al., 2012). This study, and the recently sequenced mountain pine beetle genome (Keeling et al., 2013), provides a foundation for subsequent metabolite investigation, which can further elucidate thermal cues, and levels of regulation other than transcriptional, of seasonal production of glycerol in larval mountain pine beetles.

## Abbreviations

6-PGL: 6-phosphoglucolactonase
ADH: alcohol dehydrogenase
CS: citrate synthase
DHAP: dihydroxyacetone phosphate
FBP: fructose-1,6-bisphosphatase
G3P: glycerol-3-phosphate
G3PDH: glycerol-3-phosphate dehydrogenase
G6PDH: glucose-6-phosphate dehydrogenase
GAP: glyceraldehyde-3-phosphate
GP: glycogen phosphorylase
GS: glycogen synthase
PEPCK: phosphoenolpyruvate carboxykinase
PK: pyruvate kinase
PPP: pentose phosphate pathway
TAGL: triacylglycerol lipase
TPI: triosephosphate isomerase

## Additional Information and Declarations

### Data Availability

The following information was supplied regarding data availability: Fraser, Jordie; Bonnett, Tiffany; Keeling, Christopher; Huber, Dezene (2016): Data supporting: seasonal shifts in accumulation of glycerol biosynthetic gene transcripts in mountain pine beetle, Dendroctonus ponderosae Hopkins (Coleoptera: Curculionidae), larvae. figshare.
https://doi.org/10.6084/m9.figshare.3494564.v1.
